# Making Mock-FNA Smears from Fresh Surgical Pathology Specimens to Improve Smear Preparation Technique and to Create Cytohistological Correlation Series

**DOI:** 10.1371/journal.pone.0104983

**Published:** 2014-08-14

**Authors:** Tibor Mezei, Anca Contac, Alina Iacob, Imre Egyed-Zsigmond

**Affiliations:** 1 Department of Pathology, University of Medicine and Pharmacy of Tirgu Mures, Tirgu Mures, Romania; 2 Department of Head and Neck Surgery, University of Medicine and Pharmacy of Tirgu Mures, Tirgu Mures, Romania; University of Connecticut Health Center, United States of America

## Abstract

**Background:**

Fine needle aspiration (FNA) cytology is a well-established diagnostic method based on the microscopic interpretation of often scant cytological material; therefore, experience, good technique and smear quality are equally important in obtaining satisfactory results.

**Aims of Study:**

We studied the use of fresh surgical pathology specimens for making so-called mock-FNA smears with the potential of cytohistological correlation. Additionally, we studied how this process aids the improvement of preparation technique and smear quality.

**Methods:**

Cytological aspirates from 32 fresh biopsy specimens from various sites: lung (20), lymph nodes (6), and breast (6) were obtained, all with a clinical diagnosis of tumor. Aspiration was performed from grossly palpable tumors. 25G needle and Cameco-type syringe holder was used with minimal or no suction.

**Results:**

Unfixed surgical specimens provided sufficient cytological material that resulted in good quality smears. After standard processing of specimens into microscopic sections from paraffin embedded tissues, cytohistological case-series were created. No significant alteration was reported in tissue architecture on hematoxylin-eosin stained sections after the aspiration procedure. A gradual, but steady improvement was observed in smear quality just after a few preparations.

**Discussions and Conclusions:**

Our study proved that surgical specimens may be used as a source of cytological material to create cytohistological correlation studies and also to improve FNA cytology skills. The use of very fine gauge needle (25G, 0,6 mm diameter) during the sampling process does not alter tissue architecture therefore the final histopathological diagnosis is not compromised. We conclude that by using fresh surgical specimens useful cytohistological collections can be created both as a teaching resource and as improving experience.

## Introduction

Fine needle aspiration cytology or biopsy (FNAC/FNAB) is a well-established diagnostic method used in many countries as a first-line diagnostic procedure aiding preoperative management of various palpable and non-palpable lesions. The history of needle biopsy spans several centuries; however, its use as a routine diagnostic technique was established mostly during the twentieth century [1]. It is increasingly being introduced into everyday clinical practice also in our region (Mures County, Romania). Its use is well established in the management of thyroid nodules [2]–[4], and it gains more and more popularity also in other fields of medicine such as the evaluation of breast lesions, head and neck masses, and in general superficial and deep lesions [5]–[11]. Diagnosis is based on the interpretation of cytological material aspired with a small diameter needle. This might result in often scant cellularity of smears; therefore, aspiration technique and quality of smears are crucial in obtaining satisfactory results. Prior to starting clinical practice, experience may be broadened by several ways including the use of models for FNA scenario [12].

To accelerate the learning process and experience of recognizing specific cellular morphology we propose here the use of fresh (i.e. unfixed) surgical pathology specimens as a source of creating FNA smears and cytohistological correlation series. This procedure also might serve as a model for learning the basics of FNAC including sampling and smear preparation. Our main objective was to demonstrate how fresh surgical specimens may used as a source for cytological material with respect to creating cytohistological correlation case series. Additional objective was to study the usefulness of surgical pathology specimens in the practice of fine needle aspiration technique including sampling and smear preparation.

## Materials and Methods

The study was conducted in Mures County Emergency Clinic/Spitalul Clinic Judetean de Urgenta Mures, university hospital within the University of Medicine and Pharmacy of Tirgu Mures. Upon submission a signed written informed consent was obtained from all patients acknowledging that bioptic specimens removed by surgery due to therapeutic considerations might undergo additional studies. For conducting our study we did use only some parts of the bioptic specimens, without altering in any way the final histological diagnosis. Since no additional tissues/cells were removed from any patient, other than it would anyway be removed, approval of the ethics committee was not necessary. Samples presented in this study were not described nor published in other study, including parts or the whole material.

The authors of this article did not interact with any of the patients whose surgical pathology specimens constituted the main subject of this article. The identity of patients was protected at several levels. All cases were designated with an ID number, starting with one. After the cyto-histological correlation series were created only these ID numbers identified each case. From clinical data only tumor localization, size, age, sex and histopathological type was linked to the identification numbers. This way patient confidentiality was respected by anonymizing bioptic specimens. The obtained cyto-histological correlation cases were archived according to case numbers (eg. Case #1, Case #2) plus the above mentioned minimal clinical data, without any other data that could be linked to any individual (such as name, initials, date of birth, address etc.).

Fresh, unfixed, surgical biopsies, mostly whole-organs were used as the source for cytological material aspiration, prior formalin fixation. We obtained cytological aspirates from 32 fresh biopsy specimens from various sites: lung (20), lymph nodes (6), and breast (6). All organs were submitted to the pathology laboratory with a clinical diagnosis of tumor. Aspiration was performed exclusively from grossly palpable tumors. Cytological material was obtained using standard fine needle aspiration technique as described by Stanley [13]. To avoid possible tissue architecture changes, that might hinder histopathological interpretation, maximum two passes per specimen were performed.

A Cameco-type syringe holder mounted with a 10 ml three-piece syringe was used. For staining, we used two commercially available staining kits Cytocolor and Hemacolor (Merck, Darmsadt, Germany) and standard Papanicolaou stain. In each case, we used the following two gauge type needles: 23G (length 30 mm, inner diameter 0.6 mm, manufacturer Terumo Neolus, Leuven, Belgium) and 25G (length 45 mm, inner diameter 0.5 mm, manufacturer B. Braun Sterican, Melsungen, Germany).

Aspired material was spread on standard pre-cleaned glass slides (Menzel-Glaser Superfrost, Braunschweig, Germany) using the standard one-step method described by Stanley and colleagues and its modification [13]. Two fixation techniques were implied, depending on staining protocol. For Cytocolor and Papanicolaou, wet-fixation i.e., immediate immersion into 95% ethyl-alcohol, was used, and for Hemacolor, drying was used.

## Results

**Figure 1:**
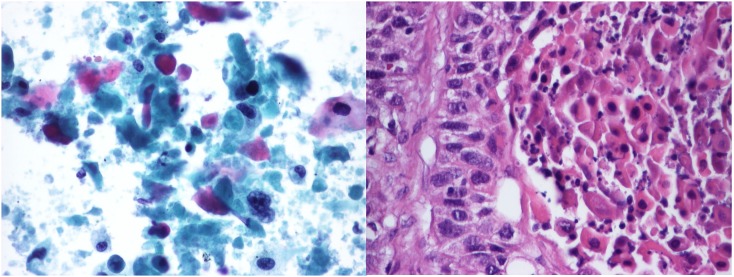
Cytohistological correlation. Left: Cytological smear (Cytocolor rapid stain, obj. magnification: 40x); Right: Histology (HE, obj. magnification: 40x). Squamous cell carcinoma of the lung, moderately differentiated.

**Figure 2:**
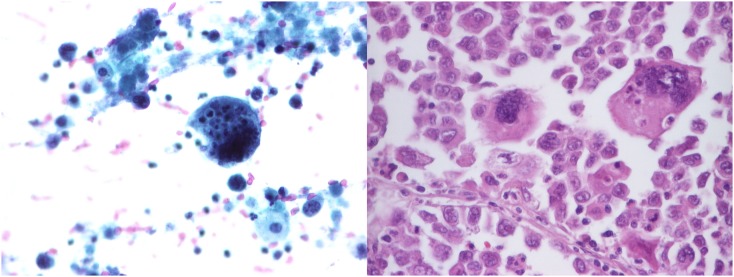
Cytohistological correlation. Left: Cytological smear (Papanicolaou stain, ob. 40x resolution); Right: Histology (HE, obj. magnification: 40x). Large cell carcinoma of the lung.

Most of the surgical specimens provided sufficient cellular quantity using the standard FNA technique. However, material obtained from lesions showing extensive desmoplasia or were rich in conjunctive stroma resulted in less cellularity. Nevertheless all cytological metrials obtained resulted in sufficient and mostly good quality smears. All the specimens were processed according to specific protocols and paraffin-embedded tissue blocks were created. Thus from all lesions microscopic slides were made and stained with hematoxylin-eosin. This permitted the creation of cytohistological correlation pairs. As depicted in Figures 1 and 2 good visuals and morphologic correlation could be made between cytological material and subsequent histological preparation from the same lesion. None of the specimens, used for aspiration, showed any significant and visible alteration, histologically.

There was no perceivable morphological differences between smears prepared with material aspired with 23G or 25G needles, both proved to be sufficent. In more fibrous or desmoplastic lesion 25G needles, handled too firmly, had a tendency to bend.

**Figure 3:**
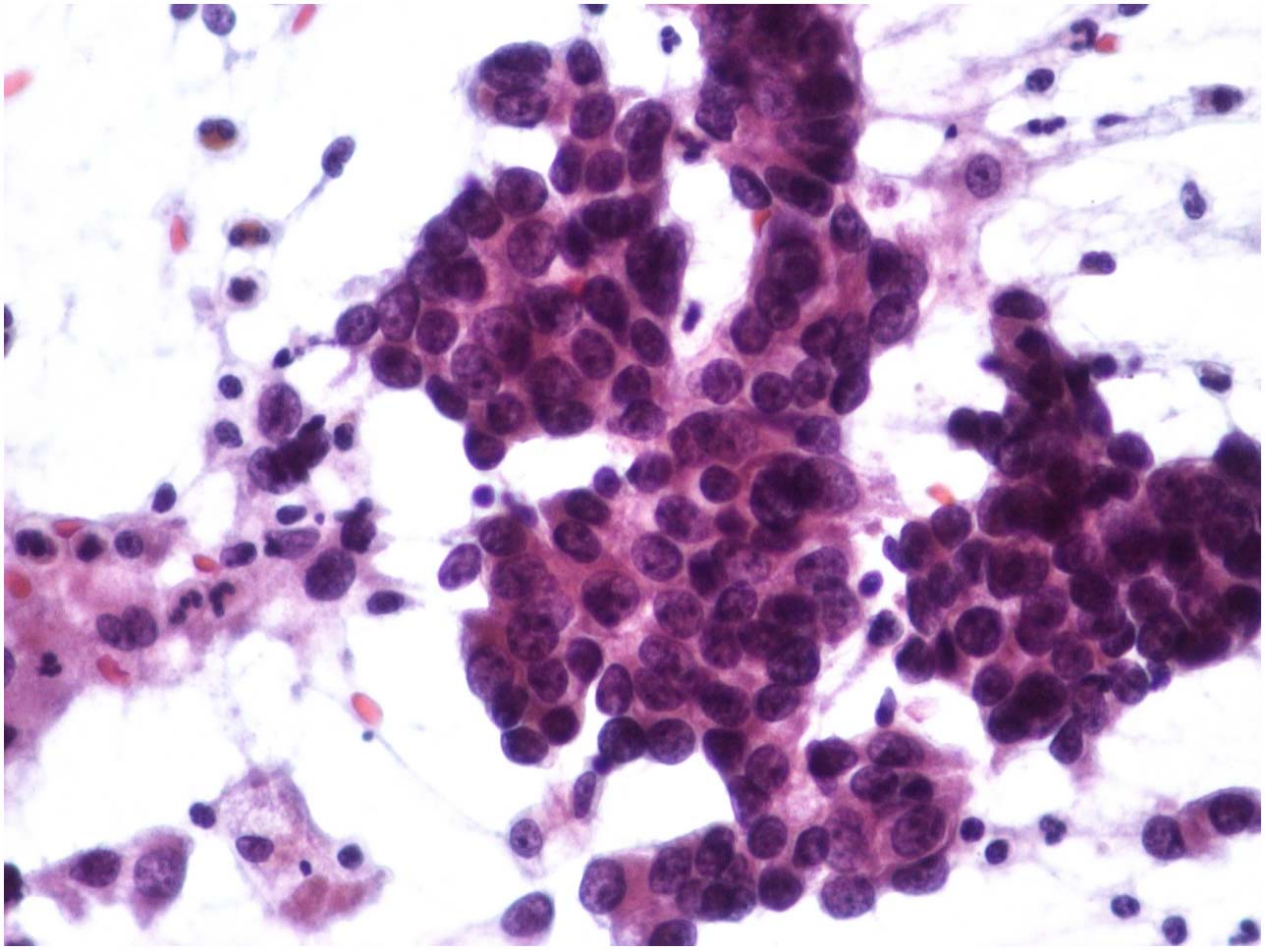
Immediately immersed smear into 95% ethyl-alcohol fixative. Staining qualities are optimal, good contrast is seen between nuclear and cytoplasmic elements (Pulmonary adenocarcinoma, Papanicolaou stain, obj. magnification: 40x).

**Figure 4:**
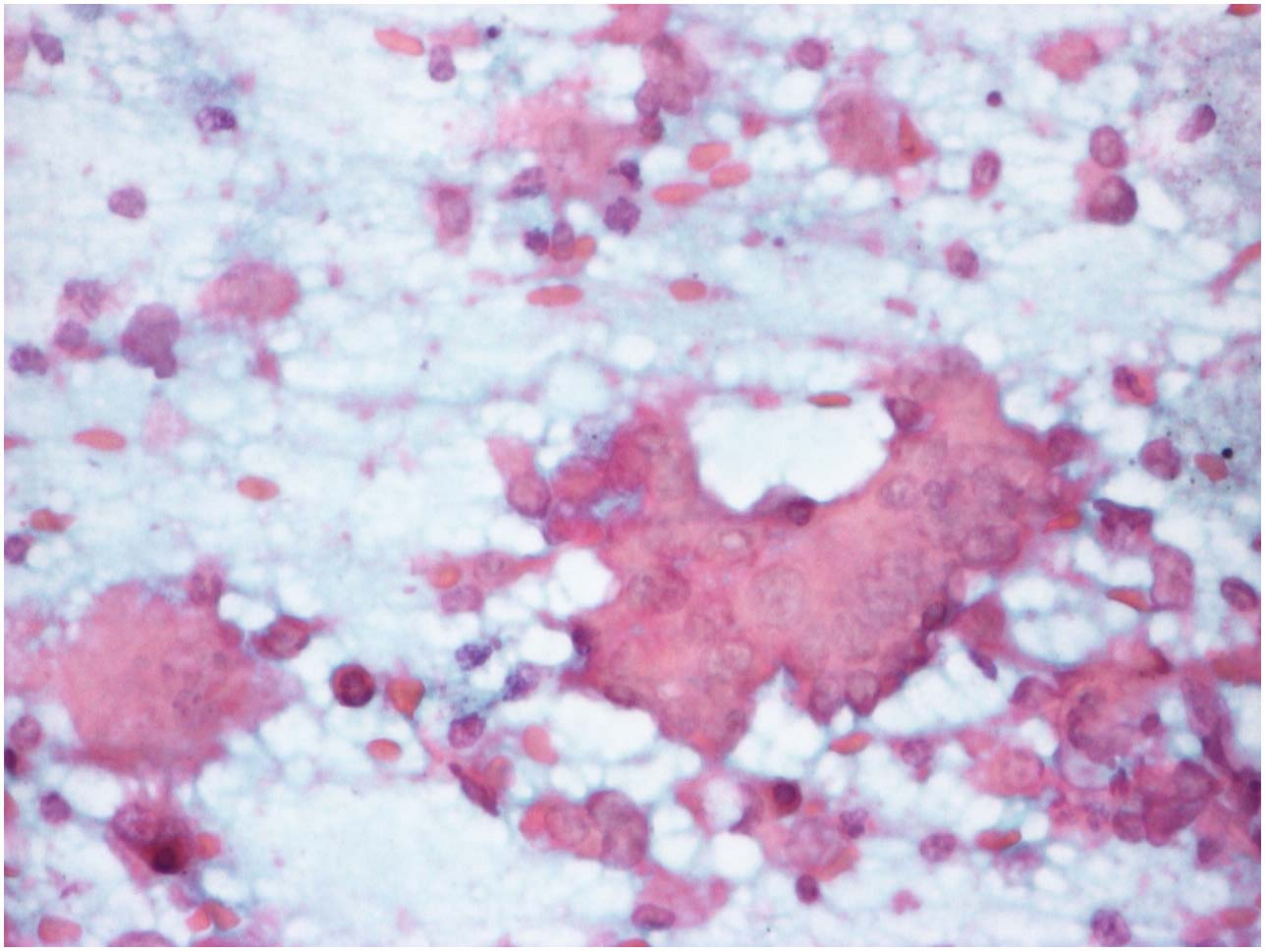
The result of delayed alcohol-fixation. The color become less vivid, contrast between nuclear and cytoplasmic staining diminishes (same case as figure 3, Papanicolaou stain, obj. magnification: 40x).

The one-step method described by Stanley prooved to be optimal in preparing cytological material obtained from fresh speciemens [13]. Slight changes in the method may cause adherence of the two slides that occurs if the spreading slide is not tilted but rather completely laid on the slide with the aspired material. Non-uniform spreading caused uneven layering of cells and sometimes collection of material at the distal end of the slides. Large quantity expulsed material from the needle caused very thick smears that created spreading and staining difficulties as well. Optimal quantity was difficult to define, but we found that 1–2 cubic mm was sufficient to enable good spreading and staining. Too large distance between the two slides caused thick cellular layer, unfit for evaluation. On the contrary, a very low distance might cause cellular, mainly nuclear, damage with consecutive artifact. The strict adherence to the method is, therefore, essential. Fixation variables (time, duration) also influenced the quality of smears. For Papanicolaou-based staining immediate fixation resulted in best result (Figure 3), delay in fixation (Figure 4) decreased drammatically the quality of smears.

## Discussion

Fine-needle aspiration cytology or biopsy (FNAC/FNAB) is increasingly performed as an economical, safe and rapid minimally invasive technique for diagnosis of various lesions including tumors and non-neoplastic lesions [14], [15]. Diagnosis based on the aspired cytological material is practically based on three fundamental elements. One is theoretical knowledge of various diseases that may occur at various body sites, this may be achieved by both mentored learning but mostly by individual study. Second is related to technique, including both aspiration and smear preparation. Third encompasses the ability to recognize, extract and extrapolate as much information from the cytological smear as possible to formulate a diagnoses that may be related to histopathological diagnosis and is clinically useful. Our study was aimed at describing a technique that may facilitate the foundation of the above mentioned second and third elements.

Using fresh surgical pathology specimens offers the possibility to experiment with various smearing and preparation techniques, the ultimate goal being to produce good quality smears. Before performing FNA at the patient’s side both dexterity and smear preparation skill may be improved by practicing on fresh surgical material without altering the histopathological interpretation of these specimens. Nevertheless there are few models described for practicing FNA technique, sampling and slide preparation. Some of these models include the use of fresh animal (beef and pork) liver introduced into a double layered latex glove, while others describe models made of silicone [16]. We describe a model that uses fresh surgical specimens that provide cytological material identical to „live” specimens. Cytological material obtained from these need the same preparatory steps, including spreading, fixation and staining as when obtaining from patients. Practicing fine needle aspiration technique from surgical specimens also greatly improve dexterity, aspiration technique, potentially reducing needle stick injuries when performing the procedure on the patient [17].

Finally, using surgical specimens also ensures that in addition to cytology, also paraffin-embedded tissue sections are also available at end of the day, enabling the development of the third foundation of cytodiagnosis, by the use of cytohistological correlation series (see figures 1 and 2).

## Conclusions

We described our experience of using surgical pathology specimens (tissue biopsies) for practicing FNAB proficiency. Fresh surgical pathology specimens, especially whole-organs may be used for training residents prior to the beginning of FNAB performance on patients in the clinical setting. Surgical specimens may be used as a source of cytological material to improve FNAB skills. These specimens also provide cytological material to study preparation conditions that may alter smear quality. We found that fresh surgical pathology specimens used prior formalin-fixation were a good source of cytological material and may be used for practicing sampling and smear preparation.

Additionally, using biopsy specimens offers the great opportunity to study cytohistological correlations. The creation of cytological smears has little impact on a laboratory’s financial expenses. No special equipment is needed and standard dyes (such as Papanicolaou and Romanowsky-types stains) may be used. For each specimen up to 3–4 cytological smears are optimal without significantly hindering grossing or delaying routine practice. Compared to standard histology additionally to glass-slides, stains and coverslips, only needles (with no larger gauge than 23–25G) and syringes (10 or 20 ml, 3-piece luer type) and fixative are needed that may be acquired at low cost. We consider that the costs of such materials are far beyond the benefits the introduction of mock-FNA into daily routine may represent.

Aspired material can also be used to study and experiment with the various conditions that may critically alter smear quality and potentially hinder the formulation of the correct diagnosis. To become proficient in sampling and smearing, it is necessary to perform many biopsies on patients. However, the process can be significantly accelerated by performing practice sessions with fresh surgical pathology specimens before attempting the procedure in the clinical setting.
